# Inhibition of *Xanthomonas fragariae*, Causative Agent of Angular Leaf Spot of Strawberry, through Iron Deprivation

**DOI:** 10.3389/fmicb.2016.01589

**Published:** 2016-10-13

**Authors:** Peter M. Henry, Samantha J. Gebben, Jan J. Tech, Jennifer L. Yip, Johan H. J. Leveau

**Affiliations:** Department of Plant Pathology, University of California at Davis, DavisCA, USA

**Keywords:** *Xanthomonas fragariae*, iron limitation, angular leaf spot, strawberry, siderophore, pyoverdine, tannic acid, nutritional immunity

## Abstract

In commercial production settings, few options exist to prevent or treat angular leaf spot (ALS) of strawberry, a disease of economic importance and caused by the bacterial pathogen *Xanthomonas fragariae*. In the process of isolating and identifying *X. fragariae* bacteria from symptomatic plants, we observed growth inhibition of *X. fragariae* by bacterial isolates from the same leaf macerates. Identified as species of *Pseudomonas* and *Rhizobium*, these isolates were confirmed to suppress growth of *X. fragariae* in agar overlay plates and in microtiter plate cultures, as did our reference strain *Pseudomonas putida* KT2440. Screening of a transposon mutant library of KT2440 revealed that disruption of the biosynthetic pathway for the siderophore pyoverdine resulted in complete loss of *X. fragariae* antagonism, suggesting iron competition as a mode of action. Antagonism could be replicated on plate and in culture by addition of purified pyoverdine or by addition of the chelating agents tannic acid and dipyridyl, while supplementing the medium with iron negated the inhibitory effects of pyoverdine, tannic acid and dipyridyl. When co-inoculated with tannic acid onto strawberry plants, *X. fragariae*’s ability to cause foliar symptoms was greatly reduced, suggesting a possible opportunity for iron-based management of ALS. We discuss our findings in the context of ‘nutritional immunity,’ the idea that plant hosts restrict pathogen access to iron, either directly, or indirectly through their associated microbiota.

## Introduction

*Xanthomonas fragariae* is the causal agent of angular leaf spot (ALS) of strawberry and an international quarantine pathogen of considerable concern to strawberry nurseries and growers (Roberts et al., 1997). Typical symptoms are watery lesions on the leaf that can become red and calloused over time (Kennedy and King, 1962). Severe infections can become systemic, causing plant collapse, and yield loss (Epstein, 1966; Hildebrand et al., 1967; Milholland et al., 1996). Control of ALS by strawberry growers is generally achieved preventatively, by planting crowns that are procured from nurseries as certified disease-free planting stock (Braun and Hildebrand, 2013). Management of *X. fragariae* in the field usually involves the foliar application of copper compounds, but due to resistance developed by the bacterium, these compounds must be applied at near-phytotoxic levels to be effective (Roberts et al., 1997; Braun and Hildebrand, 2013). Antibiotics (such as streptomycin and oxytetracycline) and induction of systemic resistance (with analogs of salicylic acid) have shown efficacy but these treatments are not broadly registered for use on strawberries, and new solutions are needed to ensure future success in management of ALS of strawberry (Roberts et al., 1997; Braun and Hildebrand, 2013).

Biological control agents (BCAs) and BCA-derived bioactive compounds provide alternative approaches to disease control (Vorholt, 2012; Zamioudis and Pieterse, 2012). Plants host an abundance of microorganisms on their leaves, and some have the ability to antagonize foliar pathogens through mechanisms such as antibiotic production, parasitism, competition for resources and space, and induced systemic resistance (Paulitz and Belanger, 2001; Elad, 2003). New bacterial and fungal BCA strains continue to be sought after, with increasing interest in those that share the same habitat as the target pathogen, so as to capitalize on habitat-specific competency (Compant et al., 2005). The observation that a BCA product such as Serenade biofungicide (active ingredient *Bacillus subtilis*) may derive its efficacy more from the bacterial production of lipopeptides during product formulation than by the activity of *B. subtilis* bacteria on the plant leaf surface (Marrone, 2002; Glare et al., 2012) has inspired efforts to derive bio-based plant protection from BCA-produced compounds rather than (or in addition to) the BCAs themselves (Glare et al., 2012). It has been suggested that this strategy might avoid some of the inconsistency that is sometimes observed in achieving adequate plant colonization by BCAs (Glare et al., 2012).

On strawberries, BCAs belonging to the bacterial species *B. subtilis* and *amyloliquefaciens*, or fungal/yeast species such as *Aureobasidium pullulans, Beauveria bassiana, Ampelomyces quisqualis*, and *Trichoderma harzianum*, have been tested in foliar applications for antagonism of strawberry pathogens, including *Botrytis cinerea* (Sylla et al., 2015) and *Sphaerotheca macularis* (Pertot et al., 2008). Several studies have also chronicled the efficacy of BCAs against species of *Xanthomonas* that are foliar pathogens on crops other than strawberry (Mishra and Arora, 2012; Naue et al., 2014; Van Hop et al., 2014). However, we are not aware of any study that has assessed BCAs or BCA-derived products in relation to *X. fragariae* on strawberry. Here, we report the serendipitous discovery of bacterial isolates from strawberry leaves with antagonistic activity against *X. fragariae* and with BCA potential. Our main objective for this study was to uncover through a series of carefully designed experiments the mechanism behind this antagonism, which we showed to be competition for iron.

## Materials and Methods

### Isolation and Characterization of *X. fragariae* and Antagonistic Strains from Strawberry

Two ALS-symptomatic strawberry plants (*Fragaria* × *ananassa* cultivar ‘Portola’) were obtained from a Northern California strawberry nursery in December 2011 and used as source material for *X. fragariae* and other strawberry leaf colonizers. Approximately 0.5 grams of infected leaf tissue (*n* = 2 from each plant) were placed in 1.5 mL centrifuge tubes and macerated in 100 μL of sterile phosphate-buffered saline (PBS). One loopful of each of the resulting suspensions was streaked in duplicate onto Wilbrink-Nitrate (WBN) agar plates (Koike, 1965) which were incubated at 20°C for 5 days. Colonies were picked from these WBN plates and re-streaked twice onto fresh WBN plates to obtain pure cultures. These so-called *Fa*P isolates (*Fa*P for *Fragaria* × *ananassa* cultivar ‘Portola’) were grown in liquid WBN medium overnight at 30°C and shaking at 250 rpm, then saved as 10% glycerol stocks at –80°C. PCR was performed using 16S rRNA gene universal primers pA and 1492r (Edwards et al., 1989) or 518r (Muyzer et al., 1993) and sent for sequencing at the UC Davis DNA sequencing was done at the UC Davis DNA Sequencing Facility using BigDye^®^ Terminator v3.1 Cycle Sequencing Kit with The Gel Company’s Better Buffer, or digested with *Btg*I, following instructions of the manufacturer (New England Biolabs). For suspected *X. fragariae* strains, we also performed Multi Locus Sequence Analysis (MLSA) of the *fyuA, gyrB, rpoD*, and 16s rRNA genes (Young et al., 2008) and PCR confirmation using *X. fragariae*-specific PCR primer sets q241, q245, and q295 (Turechek et al., 2008). Selected *X. fragariae* strains (*Fa*P21 and *Fa*P29) were tested for their ability to cause ALS symptoms by syringe-infiltrating a bacterial cell suspension in sterile Milli-Q water (OD_600_ = 0.2, corresponding to approximately 10^8^ cells/ml) into young leaves of a non-symptomatic Portola plant. Sterile Milli-Q water was used as a control. A total volume of 0.6 mL of the cell suspension was inoculated into 18 sites on each leaf (6 sites × 3 leaflets/leaf). The plants were then incubated for 22 days at room temperature under 16 h of fluorescent lighting. After 22 days, plants were assessed for ALS symptoms.

### Characterization of Bacterial Antagonists

To test bacterial strains from strawberry leaves for their ability to inhibit *X. fragariae* growth in an overlay assay, we poured onto a standard WBN agar plate 5 ml of WBN top agar (0.7%) to which were added 500 μL of a growing culture of *X. fragariae* diluted to an OD_600_ of 0.1. Plates were incubated at 20°C for 24 h, then stab-inoculated in the center of the plate with colony material of antagonistic isolates *Fa*P11-15 using a sterile toothpick. As controls, we used *P. putida* KT2440 (Nelson et al., 2002), *P. putida* 1290 (Leveau and Lindow, 2005), *Escherichia coli* TOP10 (Life Technologies), *Collimonas arenae* Cal35 (Wu et al., 2015), *Collimonas fungivorans* Ter331 (de Boer et al., 2004) and Cal2 (Uroz et al., 2014), and *Pantoea agglomerans* 299R (Remus-Emsermann et al., 2013). Plates were incubated at 20°C for 7 days at which time the diameter of the zone of inhibition was measured. In one variation of the overlay experiment, FeSO_4_ was added to the WBN agar and top agar at a final concentration of 1.4 mM. In other cases, instead of stabbing the agar with cell culture, a 0.8-cm diameter Whatman filter disk with 5 μL of 5.87 mM tannic acid (1%), 100 mM pyoverdine, or 10 mM dipyridyl solution was placed in the center of the plate on the top agar. In some experiments, a 5-μL aliquot of 1 mM pyoverdine was deposited on pyoverdine-negative mutants of *P. putida* KT2440 (see below) to test for complementation of this deficiency.

### Construction and Screening of a *P. putida* KT2440 Random Insertion Transposon Library

Electrocompetent cells of *P. putida* KT2440 were prepared by making a 1:100 dilution from an overnight culture into LB broth and growing the cells at 30°C with shaking at 275 rpm to an OD_600_ between 0.475 and 0.575 (approximately 2–2.5 h). Cell cultures were then centrifuged for 5 min at 4,200 × *g*, washed three times and resuspended in 10% glycerol. One-hundred microliter cells and 1 μl of EZ-Tn5 <KAN-2> Tnp Transposome (Epicenter) were mixed in a cold 0.2-cm electroporation cuvette, held on ice for 10 min, electroporated in a Gene Pulser Xcell Microbial System (Bio-Rad, Hercules, CA, USA) using the manufacturer recommended settings (25 μF, 200 Ω, 2500 V) and immediately added to 1 mL of SOC medium (2% Tryptone, 0.5% Yeast Extract, 10 mM NaCl, 2.5 mM KCl, 10 mM MgCl_2_, 20 mM Glucose). The cells were then placed in a shaker at 30°C and 275 rpm for 2 h and 50-μL aliquots of serial dilutions were spread onto LB agar plates supplemented with kanamycin at a final concentration of 50 mg/L (Km50). Plates were incubated at 28°C for 24 h. Individual transformants were re-streaked on LB Km50, then transferred in groups of 12 to single WBN overlay plates containing *X. fragariae Fa*P29 as the indicator strain in the overlay. Wild-type *P. putida* KT2440 served as a positive control on each plate. Mutants that failed to generate a zone of inhibition were tested three more times to confirm loss of ability to inhibit growth of *X. fragariae* in the overlay assay, before their genomic DNA was isolated using a Blood and Tissue Kit (Qiagen), digested with *Pst*I (New England Biolabs), self-ligated with T4 DNA ligase, and used as template DNA in a PCR with primers Kan-2 FP1 and RP1 (Epicenter). Amplicons were sent for sequence analysis of the DNA regions flanking the transposon insertion site. DNA sequences were mapped to the publicly available genome sequence of *P. putida* KT2440 (GenBank accession number AE015451).

### Quantification of Growth Suppression

Microtiter plate reader experiments were conducted on a Biotek Synergy 2 Multi-Mode Plate Reader in 96-well plates. *X. fragariae Fa*P29 cultures were grown on WBN to an OD_600_ of 0.2, then dispensed as 190-μL aliquots into sterile 1.5 mL Eppendorf tubes. Tannic acid (>97% purity, synonym: pentagalloyl glucose, gallotannin) was purchased from MP Biomedicals (Burlingame, CA, USA), dissolved in sterile de-ionized water, and added in 10-uL aliquots to the Eppendorf tubes to achieve final concentrations of 0.011, 0.022, 0.045, 0.9, 0.18, 0.36, 0.73, 1.46, 2.93, 5.87, 11.75, 23.5, 47, and 94 μM. Pyoverdine (>90% purity, isolated from *P. aeruginosa*) was purchased from Sigma Aldrich (St Louis, MO), suspended in sterile de-ionized water, and added to tubes for final concentrations of 0.01, 0.17, 0.34, 0.68, 1.36, 2.37, 5.46, 10.93, 21.87, 43.75, and 87.5 μM. Tubes were mixed thoroughly by pipetting before transferring the 200 μL from each Eppendorf tube to individual wells on a microtiter plate. For each concentration of tannic acid or pyoverdine, we used 4 replicate wells. In some repetitions of the experiment, FeSO_4_ was added to attain a final concentration of 20 μM at the time of aliquoting into the 96-well plate. In control wells, we added pyoverdine, tannic acid, or FeSO_4_ to uninoculated broth; readings from these wells were subtracted from the readings of their inoculated counterparts. Microtiter plates were incubated at 25°C and for every well the OD_600_ was measured every 15 min for a total of 22 h.

### Co-inoculation of Strawberry Plants with *X. fragariae* and Various Concentrations of Tannic Acid

*Xanthomonas fragariae Fa*P29 cells were grown in WBN broth to an OD_600_ of 1.0, centrifuged, washed twice in sterile de-ionized water, and resuspended in sterile de-ionized water to an OD_600_ of 1.0. This bacterial suspension was divided into three beakers and mixed with an equal volume of dissolved tannic acid to achieve final concentrations of 0, 0.003, 0.1, or 1.4 mM of tannic acid (i.e., 4 treatments). To each beaker, we added Triton X-100 to 0.0225/g/g/ to ensure even spread of bacteria and tannic acid over the leaf surface. Six plants per treatment were dipped into the corresponding beaker for 30s and leaves that were fully submerged were tagged. Plants were incubated at 28°C and disease severity was assessed 14 days after inoculation by recording the number of lesions per gram of leaf averaged per plant (three leaves per plant). This experiment was replicated three times.

## Results

### Isolation and Characterization of *X. fragariae* Strains from Strawberry

**FIGURE 1:**
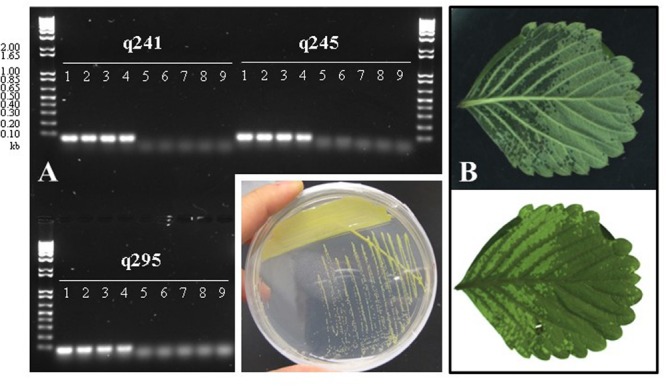
**(A)** PCR-based confirmation of *Xanthomonas fragariae* identity for selected *Fa*P isolates from ALS-symptomatic strawberry plants, using *X. fragariae*-specific primers q241, q245, and q295 (Turechek et al., 2008). Expected amplicon sizes are 60, 65, and 71 base pairs, respectively. Lanes 1: *Fa*P21, 2: *Fa*P25, 3: *Fa*P29, 4: *Fa*P33. Lanes 5 through 9 are negative controls, 5: *Xanthomonas campestris Fa*P1, 6: *X. campestris Fa*P7, 7: *Pseudomonas putida* 1290, 8: *Collimonas fungivorans* Ter331, and 9: no template control. Inset shows the characteristic yellow color of *X. fragariae* colonies on WBN agar plates. **(B)** Typical ALS symptoms (water-soaked lesions) following inoculation of strawberry with *X. fragariae Fa*P21 or *Fa*P29. Shown is the same leaf illuminated from above (top photograph) or below (bottom).

**FIGURE 2:**
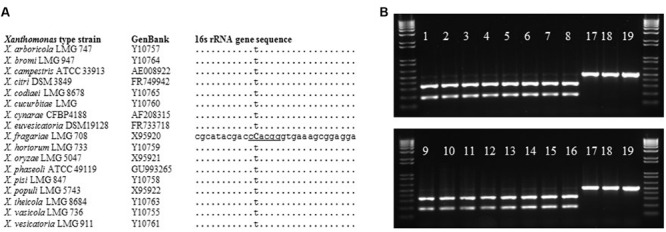
***Btg*I-based identification of *X. fragariae*. (A)**: alignment of partial 16 s rRNA gene sequences from *Xanthomonas* type strains, showing the unique cytosine (bold and capitalized) and *Btg*I recognition site (underlined) for *X. fragariae*. **(B)**: banding pattern following *Btg*I-digestion of pA-518r amplicons from the following bacterial strains: 1–16: *Fa*P21–*Fa*P36, 17: *X. campestris Fa*P1, 18: *P. putida* 1290, 19: *Collimonas fungivorans* Ter331.

In an effort to isolate *X. fragariae* strains from ALS-symptomatic strawberry plants as source material to study ALS, we followed standard procedure (Koike, 1965) by plating leaf macerates onto WBN agar plates and selecting colonies with a bright-yellow colony color that is characteristic for *X. fragariae* growing on these plates (**Figure 1**, inset). 16s rRNA gene sequencing confirmed that most isolates were indeed *X. fragariae* (e.g., isolates *Fa*P21-36). However, some were not and showed greater relatedness to other *Xanthomonas* species, i.e., *X. campestris* (e.g., isolates *Fa*P1 and *Fa*P7). Closer analysis of the 16s rRNA gene sequences of *X. fragariae* and other *Xanthomonas* species in the Ribosomal Database Project^1^ revealed a single nucleotide difference in position 187 (*E. coli* reference numbering) (**Figure 2A**). This nucleotide substitution allowed specific recognition of *X. fragariae* by digestion of the 16S rRNA gene with the enzyme *Btg*I (recognition sequence: CCRYGG; where C is a T in other xanthomonads). Indeed, PCR amplification using universal bacterial primers pA and 518r (Muyzer et al., 1993), and subsequent digestion with *Btg*I yielded the expected 179- and 323-bp fragments for *X. fragariae* isolates *Fa*P21-36, whereas the corresponding amplicons from strawberry leaf isolates *X. campestris Fa*P1 and *Fa*P7, as well as a selection of non-xanthomonads did not cut (**Figure 2B**). We further confirmed *X. fragariae* identity of a subset of *Fa*P isolates (1) by PCR analysis using *X. fragariae*-specific primers q241, q245, and q295 (Turechek et al., 2008) (**Figure 1A**), (2) by *X. fragariae*-specific MLSA (Young et al., 2008) revealing 100% identity with published *fyuA, gyrB, rpoD*, and 16s rRNA gene sequences from *X. fragariae* ATCC 29076 (EU498875, EU498979, EU499098, and X95920, respectively), and (3) for the ability to produce ALS symptoms (**Figure 1B**) after leaf inoculation of strawberry plants (*Fragaria* × *ananassa* cultivar ‘Portola’). One of these confirmed isolates, *X. fragariae Fa*P29 was selected for further experiments.

### Isolation and Characterization of Bacterial Antagonists of *X. fragariae*

**FIGURE 3:**
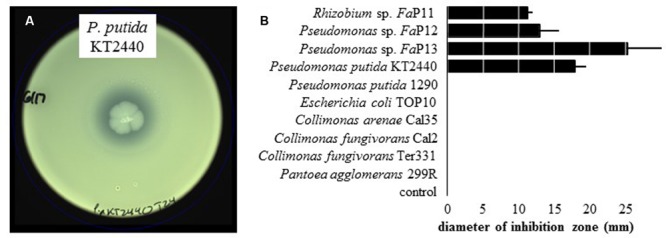
**Inhibition of *X. fragariae* by bacterial antagonists. (A)**: WBN agar overlay plate showing the absence of *X. fragariae* growth near the point-inoculated colony of *P. putida* KT2440 in the center of the plate, after 22 h of incubation. **(B)**: shown is the average diameter of the zone of *X. fragariae Fa*P29 inhibition produced by the bacterial strains listed, after 22 h. Error bars represent the standard deviation showing the variation between three independent experiments.

On WBN plates that were spread with leaf macerate from ALS-symptomatic plants to isolate *X. fragariae*, we occasionally observed growth inhibition of bright-yellow *X. fragariae* by nearby, whitish colonies. Analysis of five randomly picked colonies through partial sequencing of their 16S rRNA genes revealed that three belonged to the species *P. koreensis* (*Fa*P12, *Fa*P14, and *Fa*P15), one to *P. mandelii* (*Fa*P13), and one to *Rhizobium radiobacter* (*Fa*P11). The antagonistic activity of three of these strains (*Fa*P11, *Fa*P12, and *Fa*P13) was quantitatively confirmed in agar overlay assays (see Materials and Methods) showing a clear zone of *X. fragariae Fa*P29 inhibition around the point of antagonist inoculation (**Figure 3**). We tested several other strains in our culture collection: of these, only *P. putida* KT2440 showed antagonistic activity (**Figure 3**).

### Transposon Insertion Mutants of *P. putida* KT2440 Unable to Inhibit *X. fragariae* Growth

**FIGURE 4:**
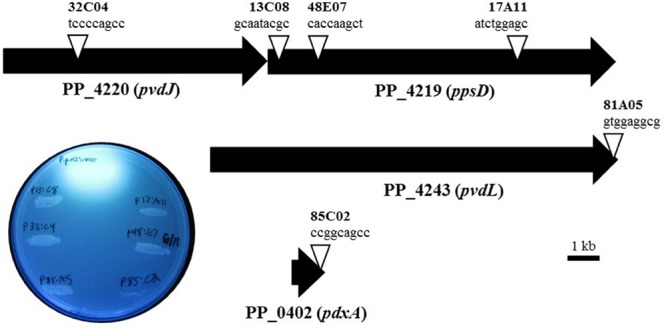
**Characterization of transposon mutants of *P. putida* KT2440 unable to inhibit *X. fragariae*.** The six triangles represent the transposon insertion sites in four genes (PP_4219, _4220, _4243, and _0402) on the KT2440 genome. For each insertion site, the insertion sequence and corresponding mutant label (e.g., tccccagcc, mutant 32C04) are shown. The inset shows the six mutants together with *P. putida* KT2440 on a UV-illuminated WBN agar plate to show the fluorescence produced by wildtype but not in mutants.

*Pseudomonas putida* KT2440 was chosen to elucidate the mechanism and gene(s) that are responsible for the observed phenotype of *X. fragariae* growth inhibition. The reason for choosing this well-studied strain was the availability of a genome sequence (Nelson et al., 2002), which greatly facilitated our search for candidate genes. We generated a library of approximately 2,700 random insertion mini-Tn*5* mutants of *P. putida* KT2440 and screened it to reveal six mutants (13C08, 17A11, 48E07, 32C04, 81A05, 85C2) which had completely lost the ability to inhibit the growth of *X. fragariae Fa*P29 in an agar overlay assay. In four of these mutants, transposon insertions mapped to the *ppsD* gene (PP_4219) or the *pvdJ* gene immediately upstream (PP_4220; **Figure 4**). The two other mutants of *P. putida* KT2440 (81A05 and 85C02) carried a Tn*5* insertion in PP_4243 (*pvdL*) and PP_0402 (*pdxA*), respectively. Genes PP_4219 (*ppsD*), PP_4220 (*pvdJ*) and PP_4243 (*pvdL*) are all annotated as coding for non-ribosomal peptide synthetases and they are among 26 genes with predicted roles in the biosynthesis of pyoverdine by *P. putida* KT2440 (Matthijs et al., 2009). Pyoverdines are a group of diffusible and fluorescent siderophores that represent the primary iron uptake system of many *Pseudomonas* species (Visca et al., 2006). The *pdxA* gene (PP_0402) is annotated as a 4-hydroxythreonine-4-phosphate dehydrogenase, an enzyme that is involved in vitamin B6 biosynthesis. In all six of our mutants, the ability to fluoresce on WBN was lost (**Figure 4**), which is consistent with previous reports of pyoverdine-negative phenotypes in *P. putida* KT2440 (Matilla et al., 2007; Matthijs et al., 2009).

### Competition for Iron as a Mechanism Underlying *X. fragariae* Growth Inhibition

**FIGURE 5:**
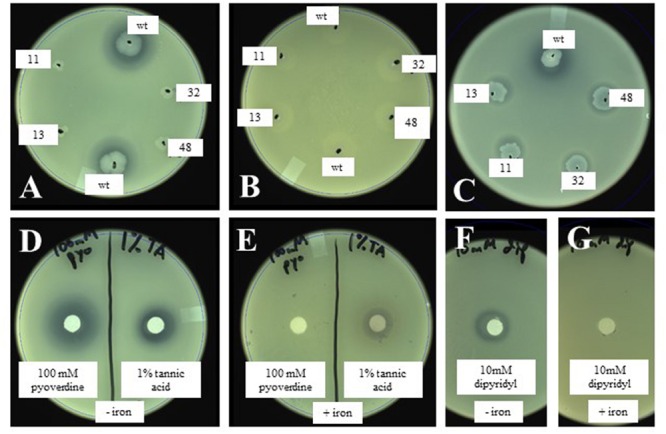
**Effect of wildtype and mutant *P. putida* KT2440 **(A,B)** and of the chelating agents pyoverdine and tannic acid **(D,E)** on *X. fragariae* growth in WBN overlays without **(A,D)** or with **(B,E)** supplemented iron (1.4 mM FeSO_4_).** Also shown is the complementation of KT2440 mutant colonies with 5 μl each of 1 mM pyoverdine **(C)**, as well at the effect of the chelator dipyridyl on *X. fragariae* growth in the absence **(F)** or presence **(G)** of supplemental iron (1.4 mM FeSO_4_). Wild-type KT2440 is labeled as ‘wt’; the four Tn*5* insertion mutants 17A11, 13C08, 32C04, and 48E07 as 11, 13, 32, and 48, respectively. Pyoverdine **(D,E)**, tannic acid **(D,E)** or dipyridyl **(F,G)** were added to a filter paper on the agar surface as 5 μl of a 100 mM, 1% (5.9 mM) or 10 mM stock solution, respectively.

Based on the above observations, we hypothesized that the inhibition of *X. fragariae Fa*P29 by *P. putida* KT2440 on WBN plates was due to iron limitation, more specifically the ability of *P. putida* KT2440 to produce the chelating agent pyoverdine and sequester iron away from *X. fragariae Fa*P29. We performed several experiments to test this hypothesis. Addition of iron (FeSO_4_) to the WBN agar overlay assay completely eliminated the antagonistic activity of *P. putida* KT2440 (**Figures 5A,B**). The same observation was made for isolates *Fa*P11-15 (**Supplementary Figure S1**) suggesting that the antagonistic activity we observed for these strains (**Figure 3**) was due to iron sequestration also. Interestingly, while *Pseudomonas* isolate *Fa*P13 turned fluorescent on WBN agar suggesting the production of pyoverdine, no such fluorescence was observed with isolate *Fa*P11; we suspect that this *Rhizobium* strain produces a chelating compound that is not pyoverdine. The deficient phenotype of selected KT2440 mutants 17A11, 13C08, 32C04, and 48E07 (**Figure 5A**) could be restored to wild-type phenotype by supplying iron to each mutant colony (**Figure 5C**). We demonstrated that *X. fragariae Fa*P29 growth was inhibited upon exposure to a commercially available preparation of pyoverdine (**Figure 5D**, left) and that this activity too was abolished with the addition of extra iron (**Figure 5E**, left). Inhibition was also seen with the chelating agents tannic acid (**Figure 5D**, right) and dipyridyl (**Figure 5F**) while addition of iron partially (**Figure 5E**, right) or completely (**Figure 5G**) restored *X. fragariae Fa*P29 growth.

**FIGURE 6:**
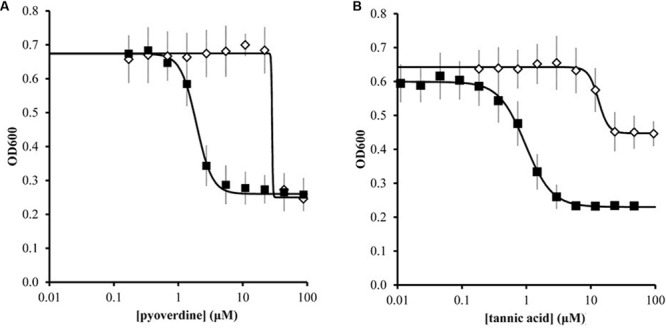
**Effect of pyoverdine **(A)** and tannic acid **(B)** on the growth of *X. fragariae Fa*P29 in liquid WBN medium.** Shown are yield, i.e., OD_600_ values measured 20 h after inoculation, as a function of chelating agent concentration. Each concentration was tested in the absence (filled squares) or presence (open diamonds) of supplemental 20 μM FeSO_4._ Error bars indicate the standard deviation of measurements from three independent experiments. Solid lines represent the best fit curves to the data using the Hill equation. These curves were used to estimate IC_50_ values. In the absence of pyoverdine (0 μM) or tannic acid (0 μM), the OD_600_ values were 0.699 ± 0.042 and 0.599 ± 0.053, respectively. In the presence of 20 μM FeSO4 but absence of pyoverdine or tannic acid, the OD_600_ value were 0.676 ± 0.067 and 0.634 ± 0.061, respectively.

We further quantified the inhibitory effects of pyoverdine and tannic acid on *X. fragariae* growth in liquid culture. For this, we measured the yield of *X. fragariae Fa*P29 (measured as OD_600_ after 20 h of growth on WBN liquid medium in a 96-well microtiter plate) as a function of pyoverdine or tannic acid concentration. From the resulting dose-response curves (**Figure 6**, filled squares), we calculated a half-maximal inhibitory concentration (IC_50_) of 1.9 μM for pyoverdine and 1.0 μM for tannic acid. Below 0.3 μM, neither pyoverdine nor tannic acid had an impact on *X. fragariae* growth, while inhibition was maximal at concentrations >5 μM. For both compounds, the maximum reduction in yield was approximately 0.35–0.4 OD_600_ units. We hypothesized that if *X. fragariae* growth was iron-limited in the presence of pyoverdine or tannic acid, the addition of iron to the WBN medium should have a negating effect on the ability of pyoverdine or tannic acid to inhibit *X. fragariae* growth. This was indeed the case: addition of 20 μM iron (FeSO_4_) increased the IC_50_ from 1.9 to 29 μM for pyoverdine-supplemented cultures and from 1.0 to 13 μM for tannic acid (**Figure 6**, open diamonds). In the presence of added iron, the impact of pyoverdine or tannic acid on bacterial growth was not noticeable until concentrations reached 20 μM or 6 μM, respectively. Maximum impact was achieved at 45 μM for pyoverdine and at 25 μM for tannic acid.

### Effects of Tannic Acid on Symptom Formation by *X. fragariae* on Strawberry

**FIGURE 7:**
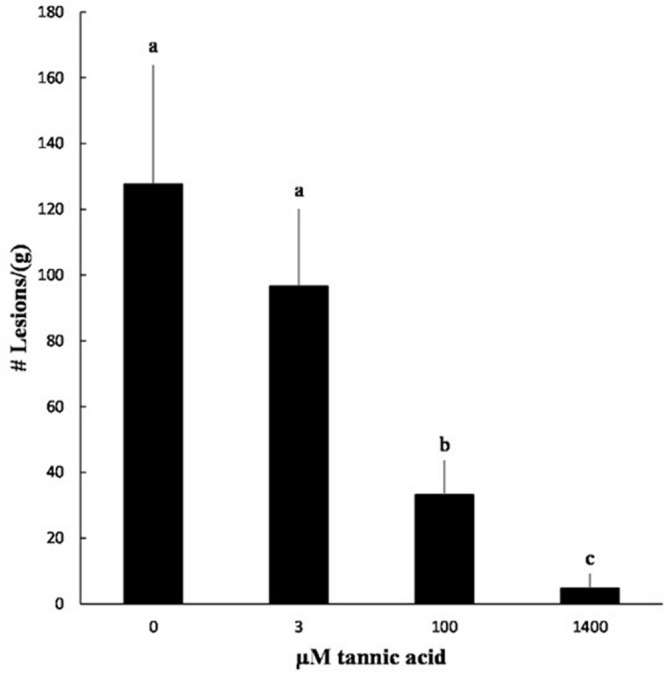
**Effect of tannic acid on symptom formation by *X. fragariae Fa*P29.** Shown is the average number of lesions per gram of strawberry leaf tissue from three independent experiments measured 14 days after foliar inoculation with *X. fragariae Fa*P29 and increasing concentrations of tannic acid. Statistical significance was determined by a *t*-test assuming unequal variance; *p*-values less than or equal to 0.05 were considered significant. Bars with the same letter represent averages that were not significantly different from each other. Error bars denote standard deviation.

We tested the ability of tannic acid to reduce symptom formation by *X. fragariae*. As a relatively cheap resource, tannic acid would offer, much more than pyoverdine, a cost-effective means of *X. fragariae* control in field settings. Co-inoculation of strawberry plants with *X. fragariae Fa*P29 and increasing concentrations of tannic acid reduced the pathogen’s ability to cause disease in a dose-dependent manner (**Figure 7**; **Supplementary Figure S2**). The lowest concentration of tannic acid that we tested was 3 μM, which also was the lowest concentration to maximally impact *X. fragariae* yield on WBN medium (**Figure 6B**), but it had no statistically significant effect on disease severity *in planta* (**Figure 7**). This was in contrast to 100 μM and 1.4 mM of tannic acid which reduced *X. fragariae* symptoms by 3.5- and 25-fold, respectively, compared to the ‘no tannic acid’ control.

## Discussion

Our results suggest that bioavailability of iron is a previously unrecognized Achilles heel of strawberry pathogen *X. fragariae*. We showed that chelating agents such as pyoverdine (in purified form or bacterially produced), tannic acid and dipyridyl effectively inhibited the growth of *X. fragariae* on agar plates and in liquid cultures and that tannic acid was able to minimize the formation of ALS symptoms *in planta*.

Iron is the most abundant element on earth (Morgan and Anders, 1980), but due to its complexation into insoluble Fe(OH)_3_ or Fe(OH)_2_ (Neilands, 1981), the amount of readily available iron is negligible compared to the growth requirements of bacteria, fungi, plants, and other organisms. Many have evolved sophisticated approaches for acquiring bio-unavailable iron (Loper and Buyer, 1991), which includes the biosynthesis of siderophores (Neilands, 1995), i.e., secondary metabolites with high enough affinity for iron to capture it from insoluble complexes (Kobayashi and Nishizawa, 2012; Saha et al., 2012). Siderophore-mediated competition for iron is common in environments such as the plant leaf surface (Loper and Lindow, 1994; Joyner and Lindow, 2000) and has been implicated as a mechanism of disease suppression by BCAs against (foliar) plant pathogens (Buysens et al., 1996; Lee et al., 2003; Santoyo et al., 2012). Among the strongest known siderophores are pyoverdine and pyochelin which are produced by *Pseudomonas* species. Once iron is bound to pyoverdine or pyochelin, no bacterial species outside the genus *Pseudomonas* has been shown to utilize iron (Loper and Lindow, 1994), including *X. fragariae* (this study).

We showed that the addition of iron to WBN medium all but abolished the inhibitory effect of strains *Fa*P11-15 and *P. putida* KT2440 on *X. fragariae*, either on WBN agar or in WBN culture. While this supports the notion that the observed inhibition is due to competition for iron, it also means that the iron concentration in our WBN medium was apparently low enough to be pushed below the iron requirements for *X. fragariae* by addition of chelating agents such as pyoverdine and tannic acid at the concentrations that we used. The need for higher doses of tannic acid to impact *X. fragariae* activity on strawberry (**Figure 7**) would suggest that iron concentrations are higher on leaves than on WBN. We do not know the iron availability on strawberry leaves, but it is likely to be a function of two factors: the plant itself and the microbiota that associate with the leaves. It has become clear from recent studies that strawberry plants carry a large and diverse community of microorganisms on their leaves (Sylla et al., 2013; Wei et al., 2016), many of which (exemplified by *Fa*P11-15) may keep iron from other leaf colonizers. However, the strawberry plant itself also may have control over leaf surface iron availability. Analogous to the production of transferrin and lactoferrin in mammals, and conalbumin in the egg whites of birds, plants produce siderophores and polyphenols that can limit iron availability to microbial epiphytes (Mila et al., 1996; Chu et al., 2010). In many plants, foliar iron is highly influenced by the amount and type of tannins that are present on the leaf surface (Karamanoli and Lindow, 2006; Karamanoli et al., 2011). In strawberry leaves, ellagitannins constitute the major fraction of polyphenolic compounds and they have a high affinity for iron (Magalhaes et al., 2014). The quantity and composition of these hydrolysable tannins appears to be cultivar-dependent (Kahkonen et al., 2001; Oszmianski et al., 2011; Aaby et al., 2012; Gasperotti et al., 2013; Karlund et al., 2014). Agromoniin and sanguiin are the main types of ellagitannins produced by strawberry, and both contain multiple trihydroxyphenyl moieties that enable tannins to bind strongly to Fe^3+^ (Vrhovsek et al., 2012). Ellagitannins have been shown to accumulate upon foliar application of benzothiadiazole (Hukkanen et al., 2007) or the pathogen *Colletotrichum fragariae* (Mamani et al., 2012). Benzothiadiazole is a plant defense elicitor compound that activates systemic acquired resistance (SAR; Hukkanen et al., 2007; Guerrero-Molina et al., 2014; Karlund et al., 2014), which in turn provides protection against a number of foliar pathogens, including *X. fragariae, S. macularis, B. cinerea* and *C. acutatum* (Terry and Joyce, 2000; Hukkanen et al., 2007; Merteley, 2010; Grellet-Bournonville et al., 2012; Braun and Hildebrand, 2013). Interestingly, infection with *X. fragariae* was recently found to decrease concentrations of ellagitannin and gallotannin in strawberry leaves (Kim et al., 2016) and it is possible that suppression of the synthesis of these compounds is a key virulence mechanism (Ishikawa et al., 2014).

The ability of tannic acid to inhibit the growth of *X. fragariae* (**Figures 5C** and **6B**) and other microorganisms (Scalbert, 1991; Chung et al., 1998) may not be solely due to its chelating properties. Other modes of action that have been proposed include astringency (i.e., enzyme inhibition and substrate deprivation) and inhibition of oxidative phosphorylation (Scalbert, 1991). To achieve a significant reduction in ALS symptoms by tannic acid, minimal concentrations of 100 μM (0.17 g per liter, or 170 ppm) were required (**Figure 7**). It is likely that at these higher doses, iron deprivation is not the only explanation for the inhibition of growth and disease-causing activity by *X. fragariae*. Notwithstanding, tannic acid performed well as a foliar treatment against *X. fragariae* in our experiments. As a relatively cheap, water soluble, ‘generally recognized as safe’, plant-derived and renewable resource, it offers potential as a novel type of foliar spray to manage *X. fragariae* in field settings. Future trials will be needed to address this potential and to optimize application dosage and frequency, and efficacy in combination with other foliar sprays.

The work on which we presented here has exposed a vulnerability in *X. fragariae* biology that may be exploited in the search for novel strategies to manage ALS. This vulnerability is related to *X. fragariae*’s dependency on its host for iron. Plants may withhold essential metals from pathogens (Fones and Preston, 2013) in a phenomenon that has been coined ‘nutritional immunity’ (Hood and Skaar, 2012). Such withholding may be direct, i.e., through host-produced compounds (Miethke and Marahiel, 2007) such as tannins in the case of plants (Mila et al., 1996), or it may be indirect, through non-pathogenic members of the plant-associated microbial community that produce chelating compounds such as siderophores. For ALS of strawberry, this opens up several new avenues in terms of exploring options to manage this disease, including but not limited to breeding of strawberry cultivars with naturally high levels of tannin, the use of elicitors to induce tannin production in plants, the foliar application of BCAs with superior iron-chelating properties, or direct application of plant- or microbially derived chelating agents.

## Author Contributions

JL conceived the study; PH, SG, JT, and JY performed the experiments and/or analyzed the data; JL and PH wrote the manuscript with edits from SG and JT.

## Conflict of Interest Statement

The authors declare that the research was conducted in the absence of any commercial or financial relationships that could be construed as a potential conflict of interest.

## Footnotes

1 https://rdp.cme.msu.edu

## Supplementary Material

FIGURE S1 **Effect of representative strawberry isolates *Fa*P11 and *Fa*P13 on *Xanthomonas fragariae* growth in WBN overlays without **(A)** or with **(B)** supplemented iron (1.4 mM FeSO_4_).** Shown are composite images of plates under visible (left) or UV (right) light. Note the difference in fluorescence on WBN plates without supplemented iron between *Fa*P11 (not fluorescent) and *Fa*P13 (fluorescent), suggesting a pyoverdine siderophore produced by *Fa*P13 but not *Fa*P11.

FIGURE S1 **Representative strawberry leaves that were inoculated with *X. fragariae Fa*P29 and **(A)** 0, **(B)** 3, **(C)** 100, or **(D)** 1400 μM tannic acid.** ALS symptoms were photographed and quantified 14 days after inoculation. See main text for details.
