# Supplementary figures and images for: Inhibition of Xanthomonas fragariae, Causative Agent of Angular Leaf Spot of Strawberry, through Iron Deprivation
Source: Front Microbiol. 2016 Oct 13;7:1589. doi: 10.3389/fmicb.2016.01589 (PMC5062028; doi:10.3389/fmicb.2016.01589)

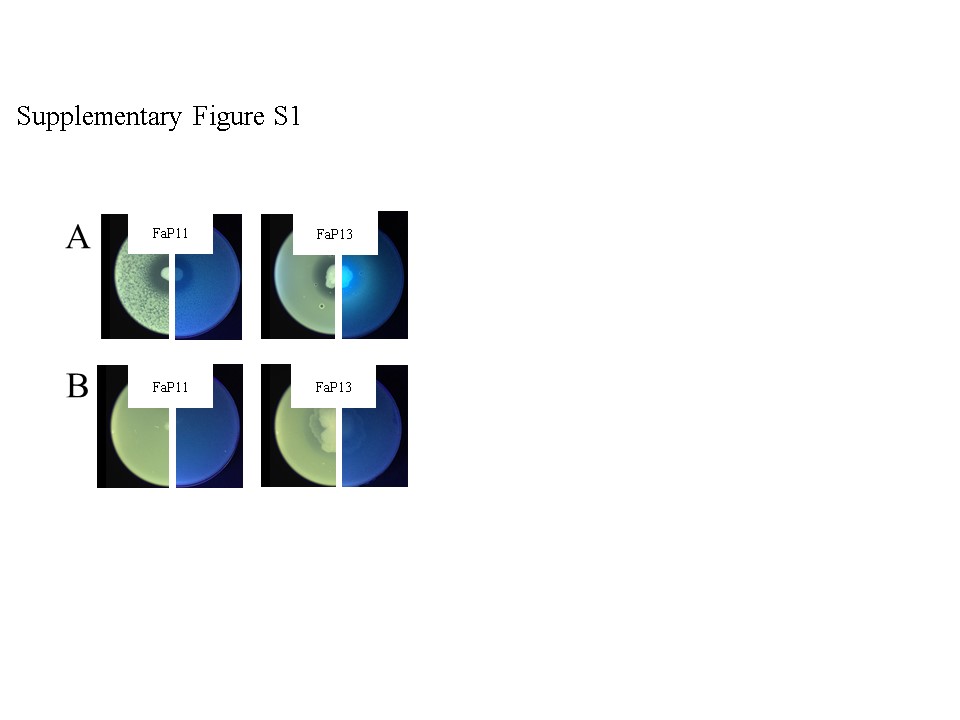

Supplement: FIGURE S1 — Effect of representative strawberry isolates FaP11 and FaP13 on Xanthomonas fragariae growth in WBN overlays without (A) or with (B) supplemented iron (1.4 mM FeSO4). Shown are composite images of plates under visible (left) or UV (right) light. Note the difference in fluorescence on WBN plates without supplemented iron between FaP11 (not fluorescent) and FaP13 (fluorescent), suggesting a pyoverdine siderophore produced by FaP13 but not FaP11. [file Image_1.JPEG]

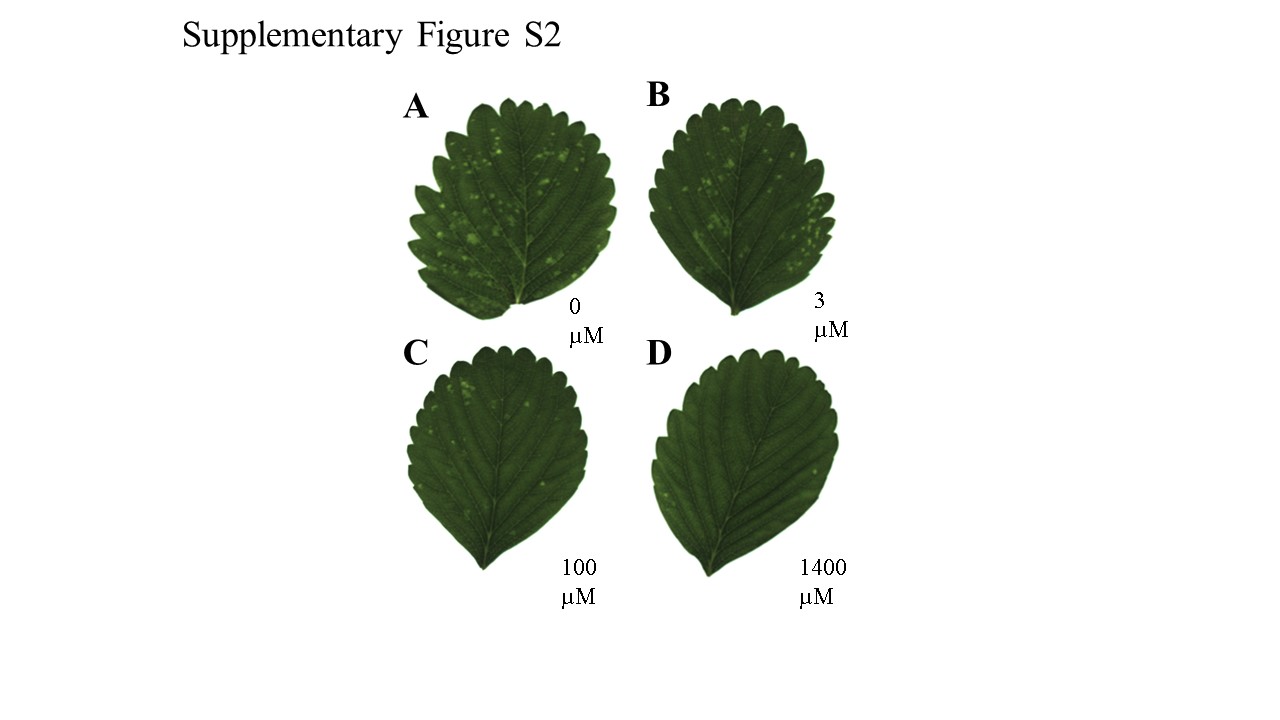

Supplement: FIGURE S1 — Representative strawberry leaves that were inoculated with X. fragariae FaP29 and (A) 0, (B) 3, (C) 100, or (D) 1400 μM tannic acid. ALS symptoms were photographed and quantified 14 days after inoculation. See main text for details. [file Image_2.JPEG]
